# Amalgam Alloys

**Published:** 1900-06-15

**Authors:** H. E. McClelland


### AMALGAM ALLOYS.

BY H. E. MC CLELLAND, D.D.S.

Read before the Odontological Society of Cincinnati, Ohio, April 27, 1900.

In taking the subject of Amalgam my object is to bring a few brief facts before this society for the purpose of invoking a discussion of this important but much-abused filling material.

I disclaim any originality but have borrowed facts from those of recognized authority.

No filling material used in the preservation of teeth has had a career so stormy as amalgam, and in spite of this opposition it is to-day saving more teeth than all other materials combined.

It was first used in Paris about 1826, and was shortly afterward introduced into this country. Had the introduction been unsuccessful from a pecuniary standpoint no doubt no notice would have been given either the material or those introducing it. But as it found favor with some of the best classes of patients, it soon began to meet with decided disfavor from the best class of practitioners of that day.

In spite of this, however, its use steadily increased, and to such an extent, that in the year 1843 it was found necessary to pronounce the use of amalgam, malpractice. This, however, did not succeed, for it still continued to grow in favor and finally the American Society of Dental Surgeons called on its members to sign a certificate pledging themselves to abandon its use, and eleven were expelled for refusing to do so.

The first practical amalgam used in this country was brought before the profession by Dr. Wm. M. Hunter, of Cincinnati, during a meeting of the American Dental Association held in this city in May 1855. The formula was silver $44\frac{4}{10}$ and tin $55\frac{6}{10}$, afterwards known as the Townsend Amalgam. Dr. Townsend, a famous dentist of Philadelphia, was most favorably impressed with it and became its chief advocate, but finally, influenced by persistent and unjust attacks against its use, abandoned it and published a letter to that effect in the *Dental News Letter* in April, 1858. Prof. Flagg was one of the first dentists to make a systematic study of the properties of amalgams and began his experiments with the Hunter formula in 1855 and gradually changed the proportions until finally he had silver the predominating material instead of tin, and it was he who recognized that the discoloration produced by the material was a virtue rather than a detriment.

The materials used at present in the manufacture of amalgam are, silver, tin, copper, gold and zinc. Silver is the dominating metal and is essential to the proper setting and maintains the bulk. It also forms the sulphids of silver which has been found beneficial in saving those teeth predisposed to decay. Tin retards setting, bulges badly, has no edge strength, but does not discolor. It greatly augments the facility of amalgamation and diminishes conductivity and is a good flux in the making of amalgams. Copper diminishes expansion and shrinkage and adds compatibility of the material to the tooth structure, and thus better preserves the teeth. When controlled by gold and zinc it aids in maintaining color. Gold controls shrinkage, increases rapidity of setting, imparts fine grain, plasticity, and secures edge strength in proper proportions of 1 to 3 percent. Zinc also controls shrinkage and adds plasticity to the mass. It also adds whiteness, but reduces strength of the alloy if more than 2 or 3 percent. is used.

Antimony, cadmium and platinium have been used to some extent but have been abandoned. Two formulas which have stood exacting tests given by Prof. Flagg, are the sub-marine used in badly decayed teeth and under the gum's margin. It is also used as a guard at the cervical margin of proximal cavities. It is composed of silver, 60 parts; tin, 35 parts and copper, 5 parts. The other, a contour alloy, used as its name implies, for contour work, maintains good color and has great strength and toughness. It is composed of silver 63⅓ parts, tin 33⅓ parts and gold 3⅓ parts. Three other formula demanding recent attention are "The Fellowship," Caulk's "20th Century" and Sibley's "Rego." They have been assayed and found to contain about the following:

| Fellowship. | | Caulk's 20th Century. | | Sibley's Rego. | |
|---|---|---|---|---|---|
| Silver, | 67.73 | Silver, | 66.81 | Silver, | 66.54 |
| Tin, | 26.33 | Tin | 27.32 | Tin | 28.14 |
| Copper, | 4.71 | Copper, | 4.39 | Copper, | 4.21 |
| Zinc, | 1.23 | Zinc, | 1.51 | Zinc, | 1.06 |

The above make very hard alloys, but owing to the large amount of silver, set very rapidly.

After selecting an amalgam that meets our requirements one of our most important considerations is the proper shaping of the cavity to receive the material. In this, many err, not taking into consideration the nature of the material.

In preparing for gold the governing principles result in making free ingress to cavities, of flush walls with antagonizing bearings, of retaining grooves and pits, of shaping the cavity slightly larger at its orifice than at base and the absence of undercuts and overhanging edges. But in preparing for amalgam the course to be pursued is far different. Cavities are made without angles and with no flush walls and few if any pits. The cavity is made larger inside than out, with concave undercuts and overhanging edges. In fact, the cavity is made as concave as possible, because of the spheroiding tendency of the material, for it must be remembered that it draws from angles and straight walls and that bulging and crevicing are the great objectionable features to guard against.

In preparation for gold we are taught to remove all decay possible, not endangering the pulp, the purpose being to secure a firm foundation to build upon, insuring solid packing to prevent leakage. But in amalgam this is lost sight of. The retaining edges and deep grooves make the mind of the amalgam worker easy on this score, for it has been proven that softened dentin will recalcify under amalgam, where it will not under gold.

The preparation and insertion of the material should be also given careful consideration, for here vital points are frequently overlooked.

Various methods of mixing amalgams have been tried, but the best results have been obtained by using the mortar, and using enough mercury to make a mass easily manipulated.

When the right amount of mercury has been found to make the right mix for any one amalgam the same proportion should be obtained each time by weight to give uniform results.

In placing in the cavity different methods have been used to secure homogenity of the mass, such as rubbing, tapping and malleting, but experiments have proved that tapping with the plugger each particle as inserted will give best results.

Rapidity of setting can be secured by wafering or making the final button of alloy very dry and also by burnishing tin or gold foil into the mass. In this regard gold has been found superior to tin, as the material absorbs enough tin to be a detriment to the alloy.

No one can deny that amalgams will save a class of teeth which otherwise would be lost; but it has been abused outrageously by its indiscriminate use. The original intention of its use has been entirely overlooked by many. Dr. Hunter said: "I do not advocate it to the exclusion of gold or in places where a good gold filling can be placed. But there are teeth worth saving, if only for a short time, where any filling requiring much pressure to compact would be contra-indicated, and here a plastic material is invaluable."

Dr. S. B. Palmer claims that "Failure in operations is mainly due to incompatibility of filling material to tooth tone."

Amalgam used conscientiously on these principles will prove a valuable ally to every practitioner of dentistry, and is as essential as gold, the cements and gutta-percha, in order to meet all requirements.

### DISCUSSION.

Dr. Hunter: I have used amalgam more or less in all my professional career. As the essayist says, I am satisfied it will save many teeth which can not be saved in any other way. I have made much use of Dr. Flagg's "Standard" amalgam, also of his "Submarine" amalgam, and have obtained very good results, though at best I have found any and all amalgams uncertain as to results, so far at least as appearance, later, goes. The new "Fellowship" alloy I like. It has seemed to me to be deficient in edge-strength, however. If you run it over an edge of enamel you may find that the thin shell has broken away, later. The first amalgam to be made which showed no shrinkage was the "King" amalgam, made in Pittsburg. I liked that very much, but in time the formula was changed, and I was not then so well satisfied with it.

Dr. H. A. Smith: Such a paper as this is helpful. I had a conversation with Dr. Black, recently, on this very subject. Dr. Black is always very guarded in what he says, and that fact seems to give weight to his opinions. He is a man who would sit up all night talking on dental subjects so long as he had an auditor. I asked him why he did not make an alloy, after all his investigating along that particular line. He was a trifle evasive, seeming to think he had not yet reached final and conclusive ends justifying the production of an alloy claiming pre-eminent superiority. I think the day may come, however, when we will find an alloy of his compounding on the market. He could make a fortune out of such an alloy. Dr. Black called my attention to the fact that his test amalgam fillings, made months ago, are undergoing constant change, which shows that conditions in the mouth are not alone responsible for the freaks of this material.

Dr. Hunter challenges my opinion that tube tests of filling materials are practically worthless. I have found no reason to change that opinion, expressed years ago. I think the time is not far distant when it will be demanded of manufacturers of amalgam alloys that they guarantee that they have thoroughly tested their special products. Every lot offered for the dentist's use will be required to have been tested. There will be, moreover, some definite proportion of mercury prepared for convenient use with definite amounts of the metal. I asked Dr. Black, "What do you regard as the best way to mix amalgam?" He thought, first, by use of the mortar, then by manipulation in the palm of the hand. I think the quick setting amalgams can not be properly mixed in the mortar. We need the heat of the hand. Dr. Black states that the better amalgam alloys are fused under hydrogen.

Dr. Cassidy has said we often do not use enough mercury to make a good mixture for a filling. If you use a minimum amount of mercury you will not have white filling. Mercury, you know, is a white metal, and it gives color to the filling. So with a minimum amount of mercury you will find that your filling tarnishes more easily.

Dr. Cassidy: There has been much prejudice against amalgam on account of the supposed poisonous effect of the mercury contained in it. But mercury, *as* mercury, is not poisonous. It may be taken into the stomach without injury. Ladies swallow small quantities of mercury with impunity for its supposed effect upon the complexion. It passes throughout the alimentary canal unchanged. ("Mercury, *after it is absorbed*, has a decided effect upon the blood, destroying the red corpuscles."—*Gorgas, Rep.*)

Dr. Hunter: I regard the testing of amalgams by tube-filling as useful in determining the comparative shrinkage of amalgams as compared one with another.

Dr. McLaughlin: I know of a case where a gold crown was inserted in close proximity to an old amalgam filling, and after a while the gold became noticeably tarnished, as if by mercury. The filling was several years old. After a time the entire crown became blackened. How is that accounted for?

Dr. Cassidy: It is not impossible that, through polarization of the different metals of the amalgam and the gold, an atom of mercury escaped and contaminated the gold.

Dr. Rose: Is the former idea that packing amalgam with ball burnishers is productive of superior results, still maintained?

Dr. Lang: I have been using "Fellowship" amalgam and "Triumph" alloy in the same mouth. The fillings, after a year, appear much alike. Both are in excellent order. I sometimes have warmed partly set pieces of amalgam, in order to soften them and make them malleable. Is a filling so made liable to fail?

Dr. Heise: I should be inclined to doubt whether such a procedure was a safe one.

Dr. Kumler: I feel, whenever I mix amalgam, the need of a definite percentage of mercury. As regards re-heating of partly hardened scraps of amalgam, I should fear disturbance of the intimate relationship of the particles, which might defeat the purpose of making the filling. I still have a definite use for copper amalgam in certain descriptions of decay of wisdom teeth.

Dr. Heise: Last summer I spent several weeks with Dr. Black. We talked about dentistry and especially about quick-setting amalgams. From all that has been said here this evening it might be inferred that Dr. Black is a strictly amalgam man; but he is not. He has use for gold as a filling material, as all of us have. We need to be constantly reminded that at the best amalgam has been and still remains an uncertain material for the purpose of filling, and that gold still holds a leading position as the filling material *par excellence*. Some one has said that under amalgam we often have a formation of secondary dentin where we would not have it under gold. I doubt that. Amalgam is not an easy material with which to make a perfect filling. Often, after all painstaking, when you come to finally trim and polish an amalgam filling, you will find some point where you have failed to adapt it perfectly to the edge of the enamel. It is then difficult to repair the fault. Now with gold we know an absolutely perfect juncture with the enamel may be made if care be used. Dr. Bonwill came pretty near to making perfect amalgam fillings. He got at his facts in a practical way, not theoretically. He fixed his matrices in position, not by means of screws or wedges of wood or other like uncertain ways, but by warming gutta percha or modeling compound so as to press it around the adjoining teeth and hold the matrix.

We should carefully discriminate in the selection of cases for using amalgam. In the mouth of a decided brunette you may allow an amalgam filling to show, with at least the assurance of its attracting less attention than it would in the mouth of a decided blonde. If the filling is to receive a brilliant polish, it had better be in the brunette's mouth than in that of the blonde.

Dr. Sage: I have noticed the lack of edge-strength of the "Fellowship" alloy of which Dr. Hunter has spoken. On one occasion I attempted to face a well-hardened filling of this alloy with gold, but it was so brittle as to break entirely away when I attempted to cut retaining grooves or pits in it. I had to remove it.

Dr. Cassidy: A free use of mercury when mixing your amalgam might possibly obviate that trouble.

Adjourned.

